# Beta-adrenergic activation induces cardiac collapse by aggravating cardiomyocyte contractile dysfunction in bupivacaine intoxication

**DOI:** 10.1371/journal.pone.0203602

**Published:** 2018-10-01

**Authors:** Jun Li, Ran Duan, Yingying Zhang, Xin Zhao, Yanxin Cheng, Yongxue Chen, Jinge Yuan, Hong Li, Jianping Zhang, Li Chu, Dengyun Xia, Senming Zhao

**Affiliations:** 1 Pain Medicine Center, The Third Hospital of Hebei Medical University, Shijiazhuang, Hebei, China; 2 Department of Anesthesiology, Hebei North University, Zhangjiakou, Hebei, China; 3 Hepatopathy Department, The Third Hospital of Hebei Medical University, Shijiazhuang, Hebei, China; 4 Department of Anesthesiology, Handan Center Hospital, Handan, Hebei, China; 5 Department of Pharmacology, Hebei University of Chinese Medicine, Shijiazhuang, Hebei, China; Scuola Superiore Sant’Anna, ITALY

## Abstract

In order to determine the role of the adrenergic system in bupivacaine-induced cardiotoxicity, a series of experiments were performed. In an animal experiment, male Sprague-Dawley (SD) rats under chloral hydrate anesthesia received intravenous bupivacaine, followed by an intravenous injection of adrenalin or isoprenalin, and the electrocardiogram (ECG), left ventricular systolic pressure (LVSP), left ventricular end-diastolic pressure (LVEDP), the maximum rate of rise of left ventricular pressure (+dP/dtmax) and the maximum rate of pressure decrease (-dP/dtmax) were continually monitored. In a cellular experiment, freshly isolated adult SD rat ventricular myocytes were perfused with bupivacaine at different concentrations in the presence or absence of isoprenalin, with or without esmolol. The percentage of the sarcomere shortening (bl% peak h), departure velocity (dep v) of sarcomere shortening and time to 50% of the peak speed of myocyte contraction (Tp50) was assessed by a video-based edge-detection system. In an additional experiment, Swiss mice pretreated with saline, isoprenalin, esmolol or dexmedetomidine received bupivacaine to determine the 50% lethal dose (LD50) of bupivacaine. Electron microscopy of myocardial mitochondria was performed to assess damage of these structures. To test mitochondrial reactive oxygen species (ROS) production, freshly isolated SD rat ventricular myocytes were incubated with bupivacaine in the presence of isoprenalin, with or without esmolol. First, our results showed that bupivacaine significantly reduced the LVSP and +dP/dtmax, as well as enhanced the LVEDP and -dP/dtmax (*P* < 0.05, *vs*. control, and *vs*. baseline). Adrenalin and isoprenalin induced a further reduction of LVSP and +dP/dtmax (*P* < 0.05, *vs*. before adrenalin or isoprenalin delivery, and *vs*. control). Second, bupivacaine induced a dose-dependent cardiomyocyte contractile depression. While 5.9 μmol/L or 8.9 μmol/L of bupivacaine resulted in no change, 30.0 μmol/L of bupivacaine prolonged the Tp50 and reduced the bl% peak h and dep v (*P* < 0.05, *vs*. control and *vs*. baseline). Isoprenalin aggravated the bupivacaine-induced cardiomyocyte contractile depression, significantly prolonging the Tp50 (*P* < 0.05, *vs*. bupivacaine alone) and reducing the dep v (*P* < 0.05, *vs*. bupivacaine alone). Third, esmolol and dexmedetomidine significantly enhanced, while isoprenalin significantly reduced, the LD50 of bupivacaine in mice. Fourth, bupivacaine led to significant mitochondrial swelling, and the extent of myocardial mitochondrial swelling in isoprenalin-pretreated mice was significantly higher than that compared with mice pretreated with saline, as reflected by the higher mitochondrial damage score (*P* < 0.01). Meanwhile, esmolol pretreatment significantly reduced the mitochondrial damage score (*P* < 0.01). Fifth, bupivacaine significantly increased the ROS in freshly isolated cardiomyocytes, and added isoprenalin induced a further enhancement of ROS production (*P* < 0.05, *vs*. bupivacaine alone). Added esmolol significantly decreased ROS production (*P* < 0.05, *vs*. bupivacaine + isoprenalin). Our results suggest that bupivacaine depressed cardiac automaticity, conductivity and contractility, but the predominant effect was contractile dysfunction which resulted from the disruption of mitochondrial energy metabolism. β-adrenergic activation aggravated the cellular metabolism disorder and therefore contractile dysfunction.

## Introduction

The high cardiotoxicity of bupivacaine is considered to involve a long-lasting inhibition of voltage gated Na^+^ channels. However, there is accumulating evidence suggesting that the blocking of Na^+^ channels is not the main mechanism of bupivacaine cardiotoxicity. In 1998, Sztark et al. reported that bupivacaine could directly inhibit complex I of the mitochondrial respiratory chain, thereby impairing mitochondrial respiratory function and energy generation[1]. Furthermore, bupivacaine has been shown to increase the proton permeability of the inner membrane of mitochondria and to decrease the mitochondrial membrane potential, ultimately causing mitochondrial dysfunction.

The negative effect of bupivacaine on oxidative phosphorylation decreases the ATP synthesis that is required for cardiac contractility. Eledjam et al. reported that myocardial strips exposed to bupivacaine recovered normal contractile force when incubated in a solution containing ATP[2]. Cho et al. reported that insulin could effectively resuscitate sudden-onset circulatory collapse caused by bupivacaine via shifts in myocardial energy metabolism from fatty acid catabolism to glycolysis, providing glycolytic ATP[3]. Thus, there is substantial evidence to suggest that inhibition of energy metabolism is a major explanation for bupivacaine cardiotoxicity.

Adrenalin is a first-line agent for treating cardiac arrest[4]. However, patients who have undergone bupivacaine-induced cardiac arrest are often resistant to adrenergic therapy and can develop severe pulmonary edema after receiving adrenalin[5]. Clinical case reports have noted that successful lipid-based resuscitation failed to achieve the return of effective circulation with sympathomimetics. Animal studies of bupivacaine-induced toxicity have shown that adrenalin could produce arrhythmias[6–8]. Hence, we propose that the adrenergic system plays a harmful role in bupivacaine-induced cardiotoxicity.

Bupivacaine is well known to induce depression of cardiac automaticity, conductivity and contractility, but the cardiac electrophysiologic and mechanical effects of β-adrenergic receptor stimulation is unknown. In this work, we assessed the effects of adrenergic activation on bupivacaine-induced depression of cardiac automaticity, conductivity and contractility *in vivo* and at the cellular level. We also examined the effects of isoprenalin and esmolol on bupivacaine-induced mitochondrial swelling, as well as ROS production.

Our data showed that bupivacaine induced a reversible depression on cardiac automaticity, conductivity and contractility, with contractile dysfunction as the major cause of cardiac collapse. Activation of β-adrenergic receptors enhanced the bupivacaine-induced systemic toxicity at least partly by aggravating the inhibition of mitochondrial respiration and therefore exacerbating cardiac contractile dysfunction.

## Methods

### Ethical approval of study protocol

The study protocol was approved by the Ethics Committee of the Third Hospital of Hebei Medical University (Shijiazhuang Hebei, China), including the use of chloral hydrate as an anesthetic in animal studies. All of the experimental animals, bred for research purposes, were provided by the Center of Experimental Animals of Hebei Medical University (Shijiazhuang Hebei, China). All of our experiments were performed in accordance with relevant guidelines and regulations.

### Chronotropic and inotropic effect of β-adrenergic agonists in bupivacaine-intoxicated rats

#### Drugs and animals

Forty-two male Sprague-Dawley (SD) rats weighing 200–250 g were used for this set of experiments. Animals were anesthetized with chloral hydrate (0.3 ml of 10% solution per 100 g of body weight), which has been demonstrated to have a less depressive effect on cardiorespiratory function than with other commonly used anesthetics[9–11]. A miniature pressure transducer (Chengdu Instrument Co., Chengdu, China) was inserted into the left ventricle (LV) via the right carotid artery. The heart rate (HR), left ventricular systolic pressure (LVSP), left ventricular diastolic pressure (LVDP), the maximum rate of rise of left ventricular pressure (dP/dtmax) and maximum rate of pressure decrease (-dP/dtmax) were monitored continuously, recorded and analyzed after 10 min of stabilization by using the MS4000U-1C Quantitative Recording of Biological Signals analysis system (Guangzhou Feilong Numerical Control Technologies Co., Ltd. Guangzhou, China).

All rats breathed spontaneously throughout this procedure. The SPO2 was recorded via a sensor placed at the end of the extremity. Body surface electrocardiograms (ECGs) were obtained with rats in the supine position. ECGs were recorded using three needle electrodes placed subcutaneously. The distal portions of the leads were secured in positions that approximated those of the lead II of a standard ECG. We chose to perform echocardiography with a straightforward, easily reproducible approach by capturing the whole cardiac cycle using an RM6240C multi-channel physiological signal acquisition and processing system (Chengdu Instrument Co.).

#### Experimental protocol

Twenty-one SD rats were randomly divided into three groups of seven per group. Bupivacaine was intravenously infused at 0.75 mg/kg/min for 12 min 40 sec, when the HR of rats was reduced by more than 30%. After 1 min 30 sec, 0.4 ml of saline, 6 μg/kg of adrenalin or 80 μg/kg of isoprenalin in 0.4 ml of saline was then intravenously injected, and ECG was continually recorded as described above.

Twenty-one SD rats were randomly divided into three groups of six per group. Bupivacaine was intravenously infused at 0.75 mg/kg/min for 12 min 40 sec. After 1 min 30 sec, 0.4 ml of saline, 3 μg/kg of adrenalin or 30 μg/kg of isoprenalin in 0.4 ml of saline was intravenously injected, and left ventricular hemodynamic variables were continually recorded as described above.

### Effect of β-adrenergic activation and bupivacaine on cardiac sarcomere shortening

We then tested the effect of β-adrenergic activation on bupivacaine-induced contractile dysfunction at the single cell level. Isoprenalin was used as a β-adrenergic selective activator, and the selective β-adrenergic antagonist esmolol was used to inhibit β-adrenergic activation.

#### Isolation of rat ventricular myocytes

Single ventricular myocytes were isolated from the hearts of adult SD rats by enzymatic dissociation as described by Isenberg and Klockner[12]. Briefly, the rats were anesthetized by intraperitoneal (i.p.) injection of sodium pentobarbital (50 mg/kg) and heparin (300 U/kg). Retrograde perfusion was performed on the rapidly excised rat heart on a Langendorff apparatus with oxygenated ice-cold Ca^2+^ free Tyrode’s solution via the aorta at a rate of 4 ml/min for 5 min. Thereafter, the heart was perfused with Ca^2+^ free Tyrode’s solution containing CaCl_2_ (34 mM) and collagenase II (300 mg/L) (Gibco, Invitrogen, Paisley, UK) at 37°C for 18 min. Finally, the left ventricles were removed, cut into smaller pieces and mildly buffered in Kreb’s solution. Single myocytes were harvested after filtration through a nylon mesh (pore size 200 mm) and stored in Kreb’s solution (at room temperature) for at least 1 h before gradually increasing the concentration of Ca^2+^ in the Kreb’s solution to 1.8 mM before the experiment. All experiments were implemented within 12 h after isolation.

#### Assessment of cell contractility

Shortening of ventricular myocytes was assessed by a video-based edge-detection system (IonOptix, Milton, MA, USA). Briefly, ventricular myocytes were mounted horizontally in an experimental chamber and perfused with normal Tyrode’s solution (1.8 mmol/L CaCl_2_) at 1 ml/min. A pair of electrodes was placed parallel to the cells. Cells were stimulated with 10 volts at a frequency of 0.5 Hz (2-msec duration) using a field stimulator. The real trace of sarcomere shortening, departure velocity of the contraction (dep v), relaxed sarcomere length (basal length, bl) and shortest sarcomere length during the contraction (peak) were recorded. The difference between peak and bl was treated as the sarcomere shortening. The percent shortening (bl % peak h) was calculated by using the relaxed sarcomere length as 100%.

The percentage changes from base value in the above parameters were tested individually. The test was repeated ten times with different rats, and data are expressed as the mean ± standard deviation (SD). Myocytes were chosen for the study according to the following criteria: (a) rod-shaped appearance with clear striations and no membrane blebs, (b) a negative staircase of twitch performance on stimulation from rest, and (c) absence of spontaneous contractions. Experiments were conducted at room temperature.

#### Experimental protocol

Takahashi reported that bupivacaine (0.5 to 50.0 mmol/L) increased the Ca^2+^ permeability of sarcoplasmic reticulum vesicles from rabbit skeletal muscle, an effect partially attributed to the influence on ryanodine-sensitive calcium channels[13]. To study the effects of bupivacaine at various concentrations on sarcomere shortening, eight different bupivacaine concentrations were used. Freshly isolated cardiomyocytes were perfused with normal Tyrode’s solution until cell contractions stabilized. The perfusate was then changed to Tyrode’s solution containing 5.9, 8.9, 13.3, 20, 30, 45, 67.5 or 101.5 μmol/L bupivacaine in the presence of 5.0 nmol/L of isoprenalin, with or without 50.0 nmol/L esmolol, in order to test contractile depression. Thereafter, cells were perfused with normal Tyrode’s solution to wash out the reagents and to ascertain contractile recovery. The contractile parameters were continuously recorded during perfusion.

### Transmission electron microscopy and qualitative morphological scoring

#### Drugs and animals

Male Swiss albino mice (18–24 g) were kept in plastic cages in a room at 25°C under a 12-h light–dark cycle (light on from 7 am to 7 pm). Food and water were available *ad libitum*. Mice were allowed to acclimate to these conditions for ≥3 days after arrival before being used for experiments. All experiments were performed under identical fluorescent lighting between 2 pm and 5 pm.

#### Experimental protocol

Twelve mice were randomly divided into four groups with three mice in each group. Saline, 0.1 mg/kg isoprenalin or 2.0 mg/kg esmolol was injected via the i.p. route at 10 ml/kg body weight. Five minutes after the injection, saline in the control group or 46.7 mg/kg bupivacaine in the other three groups was injected via the i.p. route. The mice were killed by cervical dislocation 3 min after the final injection. The chest was opened, and a tissue block of 1 × 3 mm was taken from the left ventricular wall and fixed with glutaraldehyde at 4°C within 1 min and stored at 4°C.

The tissue was postfixed with 1% osmium tetroxide (pH 7.4) in 0.1 M cacodylate buffer for 1 h at 4°C, and dehydration was performed with ethanol and ethanol-epon. The whole mounts were then epon-embedded overnight at 4°C, and epon-filled flat embedding molds were subsequently polymerized for 48 h at 60°C. Ultrathin sections (60–80 nm) were cut at room temperature with a Leica Ultracut UCT microtome (Leica Microsystems, Wetzlar, Germany) and mounted on nickel grids. Sections were then contrasted with 0.4% lead citrate and 3% uranyl acetate and viewed using a Hitachi H-7500 Transmission Electron Microscope (Hitachi, Tokyo, Japan). All buffers, fixatives and embedding materials for electron microscopy were purchased from the Microscope Center of Hebei Medical University (Shijiazhuang, China).

We evaluated high resolution black and white electron photomicrographs of cardiac mitochondria using the following ultrastructural injury scoring system from 0 to 5, first described by Joshi et al[14].: 0 = normal appearance, 1 = swelling of endoplasmic reticulum, minimal mitochondrial swelling, 2 = mild mitochondrial swelling, 3 = moderate or focal high amplitude swelling, 4 = diffuse high amplitude swelling, disruption of crystal membrane integrity, and 5 = high amplitude swelling with some mitochondrial flocculent densities and/or calcifications. Six photomicrographs of each heart sample depicting between 5 and 10 mitochondria each were evaluated. This scoring was performed by an experimenter blinded to the groups with extensive experience in morphological scoring and electron microscopy.

### Reactive oxygen species measurement

The freshly isolated ventricular myocytes from nine adult SD rats were plated as triplicates in 24-well plates at a concentration of 5 × 10^5^ cells/well with 500 μl normal Tyrode’s solution (1.8 mmol/L CaCl_2_) per well, with 8.9 μmol/L or 13.3 μmol/L of bupivacaine, in the presence of 5.0 nmol/L of isoprenalin, with or without 50.0 nmol/L esmolol. A pair of electrodes was placed into the wells. Cells were stimulated with 10 volts at a frequency of 0.5 Hz (2-msec duration) using a field stimulator for 1 min, and the intracellular ROS levels were evaluated by staining cells with DCFH-DA. The cells were incubated with 10 μM DCFH-DA at 37°C during the last 20 min of treatment. The cells were washed three times, collected and resuspended in PBS. Subsequently, the fluorescent signal intensity of DCF was determined by an Infinite M1000 PRO premium multimode microplate reader (Tecan Austria GmbH, Grdig, Austria) at an excitation wavelength of 488 nm and at an emission wavelength of 525 nm.

### Statistical analyses

Data were analyzed using SPSS v13.0 (SPSS, Chicago, IL, USA). The independent samples *t*-test (non-parametric statistical test for parameters of non-normal distribution) was used for intergroup comparisons. The paired t-test was used for intragroup comparisons (compared with baseline data). The intergroup differences of mitochondrial swelling scores were tested by the Kruskal-Wallis method followed by the Nemenyi test for multiple comparisons. The fluorescence intensity data were analyzed using IBM SPSS Statistics 21.0, and *P* < 5 was considered significant.

## Results

### Bupivacaine induces a dose-dependent and reversible depression on cardiac automaticity and conductivity, while adrenalin and isoprenalin can improve cardiac automaticity but not conductivity

**Fig 1:**
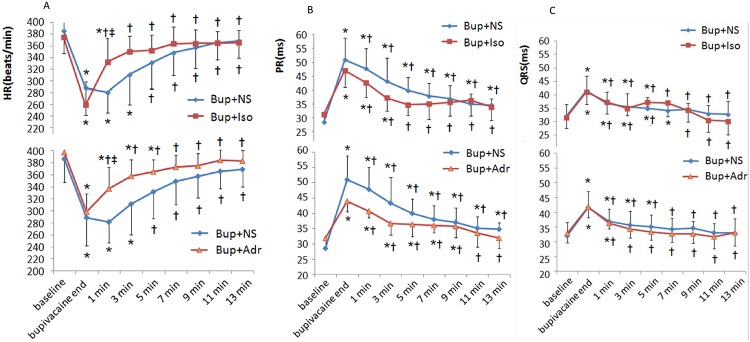
Chronotropic effect of β-adrenergic agonists in bupivacaine-intoxicated rats. (A) Bupivacaine significantly reduced HR, while adrenalin and isoprenalin restored HR. (B) Bupivacaine prolonged PR interval, while isoprenalin and adrenalin showed no effect on PR interval. (C) Bupivacaine widened QRS duration, while isoprenalin and adrenalin showed no improvement on QRS duration. *P < 0.05, *vs*. baseline; ^†^*P* < 0.05, *vs*, bupivacaine end; ^‡^*P* < 0.05, *vs*. bupivacaine + NS group.

No significant differences were observed among the groups in all parameters at baseline. The rats breathed spontaneously throughout the experiment, and the SPO2 was never lower than 90% during the experiment and not different among the groups. No rats died before the experiment was concluded. Both adrenalin and isoprenalin significantly enhanced the HR (shown in S1 Fig). Bupivacaine was intravenously infused at 0.75 mg/kg/min for 12 min 40 s, and 9.5 mg/kg of bupivacaine was delivered. All animals survived until the completion time of the experiment. As shown in Fig 1A, bupivacaine significantly reduced the HR, while adrenalin and isoprenalin restored it (*P* < 0.05, *vs*. control group, *vs*. before isoprenalin and adrenalin delivery).

As shown in Fig 1B and 1C, bupivacaine significantly prolonged the PR interval and widened the QRS duration. Isoprenalin and adrenalin showed no improvement on the PR or QRS duration (*P* > 0.05, *vs*. control group, *vs*. pre-isoprenalin or adrenalin delivery).

Bupivacaine-induced depression on cardiac automaticity and conductivity was reversible, as shown in Fig 1A–1C. After termination of the intravenous bupivacaine infusion, the HR, PR interval and QRS were totally restored within 15 min in the control group.

### Bupivacaine depresses cardiac contractility, while adrenalin and isoprenalin aggravate this depression

**Fig 2:**
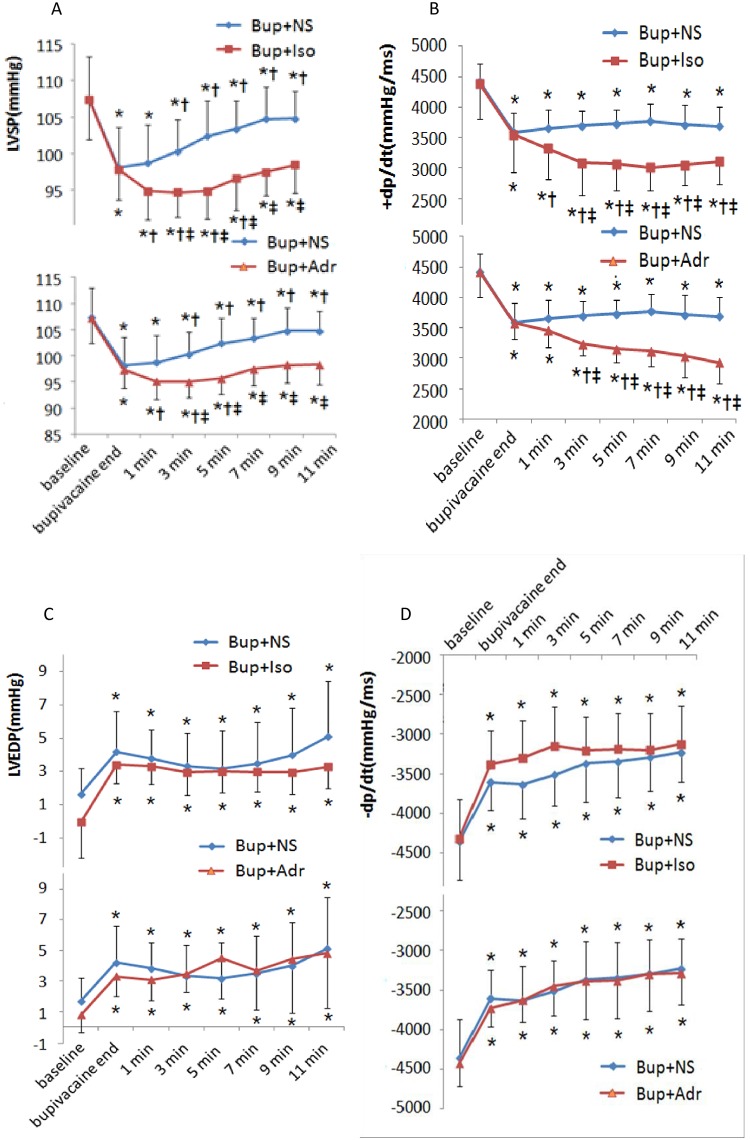
Inotropic effect of β-adrenergic agonists in bupivacaine-intoxicated rats. (A) and (B) Bupivacaine significantly reduced LVSP and +dp/ptmax, while isoprenalin and adrenalin induced a further reduction of LVSP and +dp/ptmax. (C) and (D) Bupivacaine significantly increased LVEDP and -dp/ptmax, while isoprenalin and adrenalin did not show a significant effect. *P < 0.05, *vs*. baseline; ^†^*P* < 0.05, *vs*. bupivacaine end; ^‡^*P* < 0.05, *vs*. bupivacaine + NS group.

Bupivacaine significantly impaired myocardial contractile function. As shown in Fig 2A–2D, bupivacaine significantly reduced the LVSP and +dP/dtmax and enhanced the LVEDP and -dP/dtmax (*P* < 0.05, *vs*. control). Isoprenalin and adrenalin markedly increased the bupivacaine cardiotoxicity. In the primary experiment, under anesthesia with chloral hydrate (0.3 ml of 10% solution per 100 g of body weight), 10 SD rats were equally divided into two groups, and 6 μg/kg of adrenalin or 80 μg/kg of isoprenalin was delivered at 1 min 30 sec after the bupivacaine infusion. Perhaps be due to cardiac trauma or injury caused by the transducer insertion, the animals could not tolerate the toxicity, and all 10 rats died after receiving isoprenalin or adrenalin.

In order to keep the rats alive and to exam the inotropic response, we reduced the dose of the β-adrenagic activator, and 3 μg/kg of adrenalin or 30 μg/kg of isoprenalin was used in contractile measurements. Our results showed that the β-agonist significantly worsened the bupivacaine-induced myocardiac contractile dysfunction. As shown in Fig 2A and 2B, bupivacaine significantly reduced the LVSP and +dP/dtmax, and 3 μg/kg of adrenalin and 30 μg/kg of isoprenalin induced a further reduction of LVSP and +dP/dtmax (*P* < 0.05, *vs*. before adrenalin or isoprenalin delivery, and *vs*. control). As shown in Fig 2C and 2D, the β-agonist conferred no significant effect on the LV diastolic function, and LVEDP and -dP/dtmax showed no significant difference between the bupivacaine + isoprenalin or bupivacaine + adrenalin and bupivacaine + normal saline (NS) groups (*P* > 0.05, bupivacaine + isoprenalin group *vs*. bupivacaine + NS group; bupivacaine + adrenalin group, *vs*. bupivacaine + NS group).

### Bupivacaine induces a dose-dependent cardiomyocyte contractile depression, and isoprenalin aggravates bupivacaine-induced cardiomyocyte contractile depression

**Fig 3:**
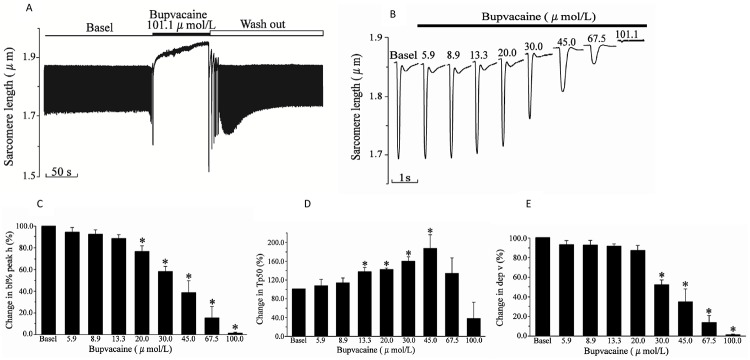
Bupivacaine mediated a concentration-dependent depression on contraction of rat cardiomyocytes. (A) Representative sarcomere-shortening trace before and after exposure to bupivacaine. Y axis: changing of sarcomere length recorded during myocyte contraction; X axis: perfusates. (B) Representative individual signal of cardiomyocyte sarcomere contraction in the presence of bupivacaine at different concentrations. (C), (D), and (E) Statistical analysis of effect of bupivacaine on bl% peak h, Tp50 and dep v. Data are the mean ± SD. *P < 0.05, *vs*. basal amplitude.

Bupivacaine induced a dose-dependent cardiomyocyte contractile depression. While 5.9 μmol/L or 8.9 μmol/L bupivacaine resulted in no change, the dose of 13.3 μmol/L bupivacaine significantly prolonged the Tp50 (*P* < 0.05). Meanwhile, 20.0 μmol/L of bupivacaine not only prolonged the Tp50, but also reduced the bl% peak h (*P* < 0.05), and 30.0 μmol/L of bupivacaine prolonged the Tp50, reduced the bl% peak h and dep v (*P* < 0.05) (Fig 3).

**Fig 4:**
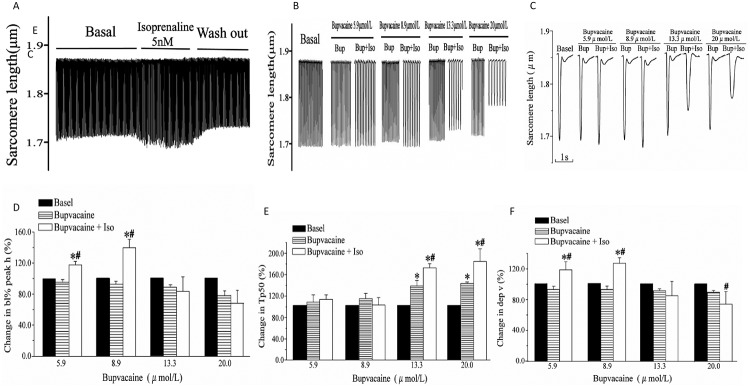
Effect of isoprenalin on bupivacaine-mediated depression of cardiomyocyte contractility. (A) Representative sarcomere-shortening trace before and after exposure to isoprenalin. (B) Representative sarcomere-shortening traces before and after exposure to bupivacaine with and without isoprenalin. Y axis: changing of sarcomere length during myocyte contraction; X axis: perfusates. (C) Representative individual signal of cardiomyocyte sarcomere shortening in the presence of bupivacaine, with and without 5.0 nmol/L isoprenalin. The signal was obtained by the averaging of ten successive signals. (D), (E) and (F) Statistical analysis of the effect of isoprenalin on bl% peak h, Tp50 and dep v in the presence of bupivacaine. Data are the mean ± SD. *P < 0.05, *vs*. basal value; ^†^*P* < 0.05, *vs*. bupivacaine group.

Fig 4 shows the inotropic effect of isoprenalin on ventricular cardiomyocytes in the presence of bupivacaine. Isoprenalin exhibited a positive inotropic effect with no or with low-concentration bupivacaine, but a negative inotropic effect with high-concentration bupivacaine. Fig 4A shows representative sarcomere shortening traces of ventricular myocytes in an isolated adult rat with isoprenalin alone. Isoprenalin increased the sarcomere shortening. Fig 4B shows representative sarcomere shortening traces of ventricular myocytes in an isolated adult rat in the presence of isoprenalin with different concentrations of bupivacaine. Fig 4C shows representative individual signals obtained by averaging ten successive signals. As shown in Fig 4D–4F, with 5.9 μmol/L and 8.9 μmol/L bupivacaine, isoprenalin significantly enhanced the bl% peak h (*P* < 0.05, *vs*. basal value; *P* < 0.05, *vs*. bupivacaine alone) and dep v (*P* < 0.05, *vs*. bupivacaine alone). However, in the presence of 20.0 μmol/L of bupivacaine, isoprenalin significantly prolonged the Tp50 (*P* < 0.05, *vs*. bupivacaine alone, Fig 4E) and reduced the dep v (*P* < 0.05, *vs*. bupivacaine alone, Fig 4F).

### Esmolol inhibits isoprenalin-induced aggravation of myocardial depression

**Fig 5:**
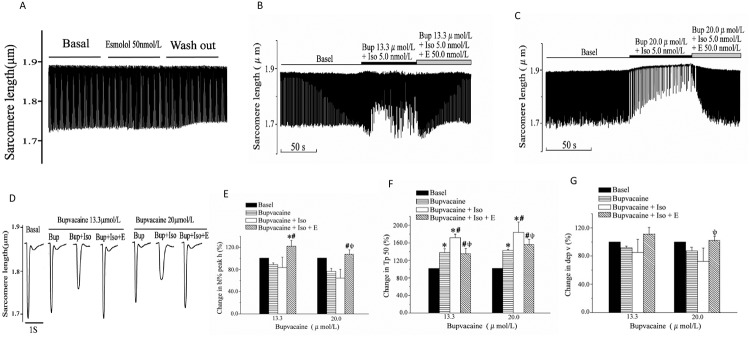
Isoprenalin aggravated bupivacaine-induced myocardial dysfunction, and esmolol inhibited the aggravation. (A) Representative sarcomere-shortening trace before and after exposure to esmolol. (B) and (C) Representative sarcomere-shortening trace perfused with bupivacaine in the presence or absence of bupivacaine + isoprenalin, with or without esmolol, in succession. (D) Averaging ten successive signals of (B) and (C). (E), (F) and (G) Effect of esmolol, isoprenalin and bupivacaine on bl% peak h, Tp50 and dep v. Data are the mean ± SD. *P < 0.05, *vs*. basal amplitude. ^†^*P* < 0.05, *vs*. bupivacaine; ^‡^*P* < 0.05, *vs*. bupivacaine + isoprenalin.

To identify the β-adrenoceptor responsible for aggravation of myocardial depression, we repeated the test in the presence of esmolol. Fig 5A shows representative sarcomere shortening traces in isolated adult rat ventricular myocytes with esmolol alone. Fig 5B and 5C show representative sarcomere shortening traces with bupivacaine + isoprenalin, and with bupivacaine + isoprenalin + esmolol in succession. Fig 5D shows representative individual signals obtained by averaging ten successive signals, and esmolol markedly reversed the effect of bupivacaine. Fig 5E, 5F and 5G show results of the statistical analysis. Esmolol ameliorated the effect of isoprenalin on the Tp50 (*P* < 0.05), bl% peak h and dep v.

### Bupivacaine-mediated contractile depression is reversible

**Fig 6:**
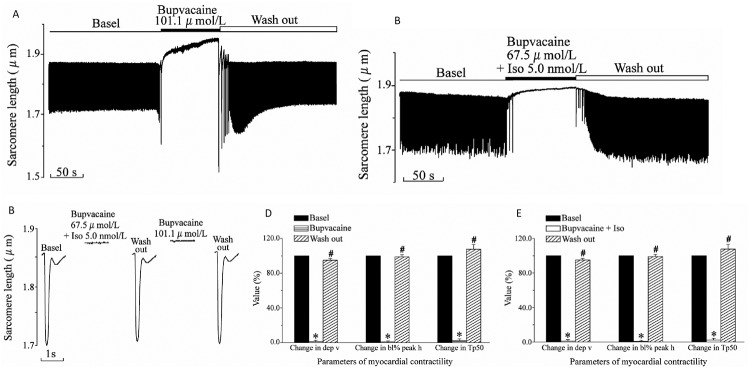
Removal of bupivacaine and isoprenalin restored cell contractility. (A) and (B) Representative sarcomere shortening perfused with bupivacaine in the presence or absence of isoprenalin, and after washing with Tyrode’s solution. (C) Individual signals of sarcomere shortening (obtained by averaging of ten successive recordings) in the presence of bupivacaine, with or without isoprenalin. (D) and (E) Statistical analysis of effect of bupivacaine on bl% peak h, Tp50 and dep v. Data are the mean ± SD. *P < 0.05, *vs*. basal amplitude; *P* < 0.05, *vs*. removal of bupivacaine and isoprenalin.

We found that 101.1 μmol/L bupivacaine, as well as 67.5 μmol/L bupivacaine + 5.0 nmol/L isoprenalin, totally inhibited cardiomyocyte contractions. However, washing out bupivacaine and isoprenalin with Tyrode’s solution restored the contractile function (Fig 6). After washing, sarcomere shortening showed the same form as that from the basal reading (Fig 6A–6C), and contractile parameters showed no significant differences compared with basal values (*P* > 0.05) (Fig 6D and 6E).

### Isoprenalin aggravates, while esmolol attenuates bupivacaine-induced myocardial mitochondrial swelling

**Fig 7:**
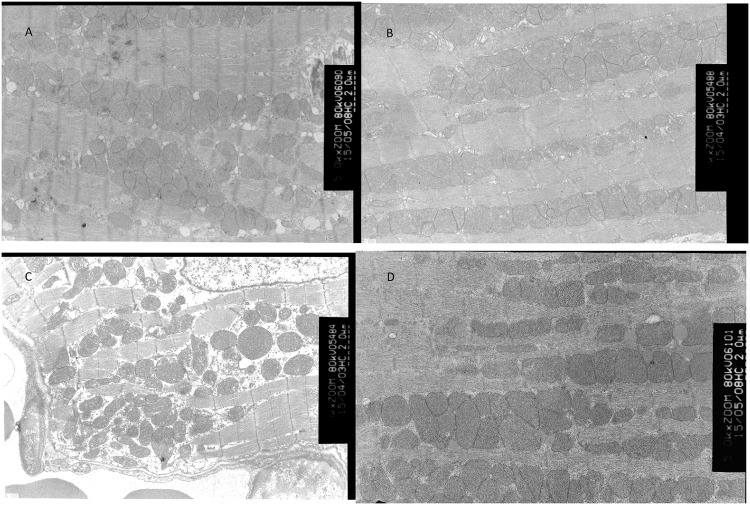
Representative electron microscopy images of cardiac mitochondria of each of the four groups. (A) Control group mice were pretreated with saline and then received saline 5 min later. (B), (C), (D) Mice were pretreated saline, 0.1 mg/kg of isoprenalin or 5 mg of esmolol, respectively, and then received 46.7 mg/kg of bupivacaine 5 min later. Arrows indicate focal high-amplitude matrix swelling.

**Fig 8:**
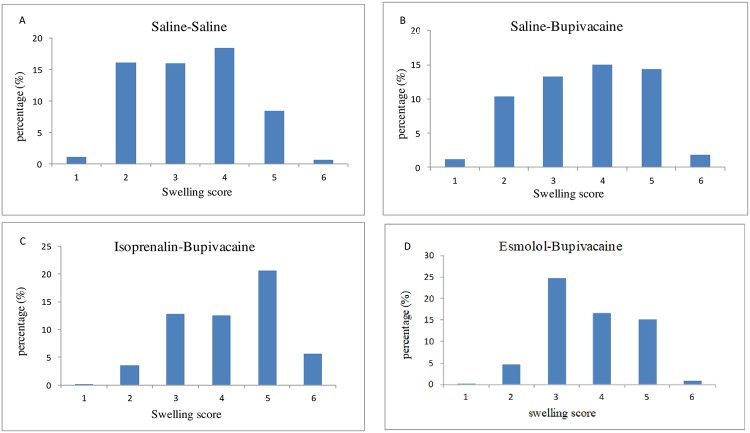
Mitochondrial damage score. Each graph depicts the mitochondrial damage score (0 = no swelling to 5 = maximum swelling) distribution in percent of n evaluated mitochondria in each group. (A) Control mice were pretreated with saline and then received saline i.p. (B), (C) and (D) Mice were pretreated with saline, isoprenalin or esmolol, and then received bupivacaine i.p. The intergroup differences of mitochondrial swelling scores were tested by the Kruskal-Wallis method followed by Nemenyi test for multiple comparisons. The order of the groups according to damage score is as follows: isoprenalin + bupivacaine group (C) > saline + bupivacaine group (B) > esmolol + bupivacaine group (D) > saline + saline group (A) (*P* < 0.01, between each two groups).

Bupivacaine led to significant mitochondrial swelling, and the extent of myocardial mitochondrial swelling in isoprenalin-pretreated mice was significantly higher than that from mice pretreated with saline, as reflected by the higher mitochondrial damage score (*P* < 0.01, saline-bupivacaine group *vs*. saline-saline group; isoprenalin-bupivacaine group *vs*. saline-bupivacaine group). Meanwhile, esmolol pretreatment significantly reduced the mitochondrial damage score (*P* < 0.01, esmolol-bupivacaine group *vs*. saline-bupivacaine group) (Figs 7 and 8). Much greater high-amplitude matrix swelling was exhibited as shown in Fig 7C compared with Fig 7D.

### Bupivacaine increases ROS production in cardiomyocytes, and isoprenalin further increases the production

**Fig 9:**
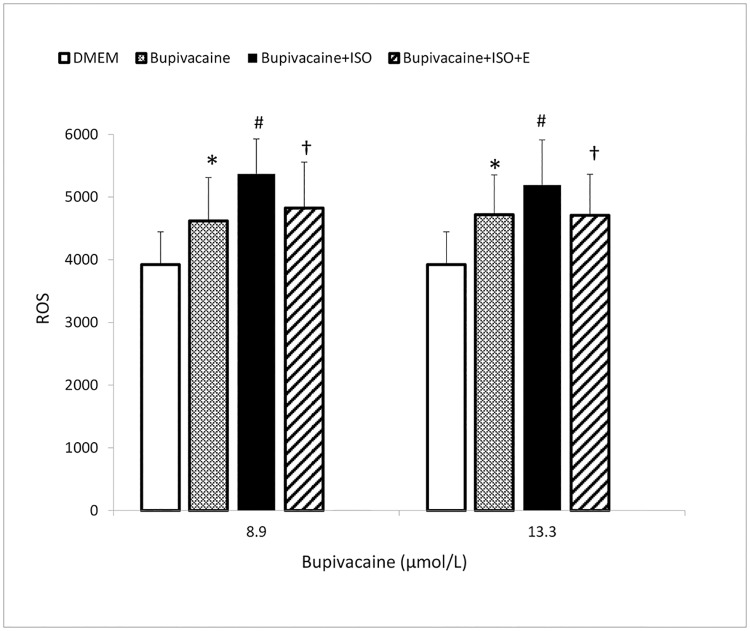
Isoprenalin enhances bupivacaine induced ROS production in freshly isolated cardiomyocytes. Saline group: cells incubated in normal saline. Bupivacaine group: cells incubated with 8.9 μmol/L or 13.3 μmol/L of bupivacaine. Bupivacaine + isoprenalin group: cells incubated with bupivacaine and 5.0 nmol/L of isoprenalin. Bupivacaine + isoprenalin + esmolol group: cells incubated with bupivacaine and 5.0 nmol/L of isoprenalin in the presence of 50.0 nmol/L of esmolol. *P < 0.05, *vs*. cells in saline; ^#^*P* < 0.05, *vs*. cells incubated with bupivacaine; ^†^*P* < 0.05, *vs*. cells incubated with bupivacaine and 5.0 nmol/L of isoprenalin.

In order to mimic the situation *in vivo*, the cardiomyocytes were electrically stimulated for 1 min during intubation with bupivacaine in the presence or absence of isoprenalin and esmolol. Under 0.5 Hz electrical stimulation, the freshly isolated cardiomyocytes showed a rhythmic contraction. As shown in Fig 9, 8.9 μmol/L and 13.3 μmol/L of bupivacaine significantly increased the ROS in freshly isolated cardiomyocyte, and added isoprenalin induced a further enhancement of ROS production induced by bupivacaine (*P* < 0.05, *vs*. bupivacaine). Added esmolol significantly decreased the ROS production (*P* < 0.05, *vs*. bupivacaine + isoprenalin).

## Discussion

Bupivacaine-induced cardiac arrest is known to be resistant to adrenergic therapy, but the underlying mechanism is still unclear. Here, we showed that β-adrenergic activation aggravated the cardiac contractile dysfunction by intensifying the cardiac mitochondrial metabolic disorder.

In order to induce cardiac toxicity, bupivacaine was intravenously infused until the heart rate of rats was reduced by more than 30%. Adrenalin is a widely accepted drug and component of cardiopulmonary resuscitation. To show the role of the β-adrenergic system in bupivacaine-induced cardiac depression, adrenalin was employed in this work. As doses higher than 30 μg/kg are associated with increased rates of myocardial dysfunction, neurological injury and a poor prognosis[15–17], only 6 μg/kg of adrenalin was used. Isoprenalin, a synthetic catecholamine and highly selective β-adrenergic agonist, has been used at 5 mg/kg for 7 days to create a rat model of cardiomyopathy[18–20]. In order to avoid isoproterenol-induced cardiac toxicity, only 80μg/kg of isoprenalin was used in our work. Our results shows that adrenalin or isoprenalin alone significantly enhanced the cardiac function of the animals.

It is well known that central nervous system effects may be involved in the production of serious cardiotoxicity. Hasselstrom *et al*. reported that intravenous bupivacaine significantly enhanced plasma catecholamine levels in humans[21]. Bernards and Artu reported that inhibiting the autonomic nerve system may suppress bupivacaine-induced cardiac arrhythmia[22]. In this work, we assessed the effect of dexmedetomidine, esmolol and isoprenalin on the LD50 of bupivacaine in mice. Our data (S2 Fig) showed that inhibition of the sympathetic system by dexmedetomidine or blocking cardiac β-adrenoceptors by esmolol significantly enhanced, whereas activation of the receptors by isoprenalin reduced, the mice of tolerance to bupivacaine toxicity. Moreover, our cellular studies showed that β-adrenergic activation markedly worsened the contractile dysfunction. Accordingly, we propose that the “indirect cardiotoxicity” of bupivacaine resulted mainly from sympathetic system over excitation. The endogenous adrenalin/noradrenalin worsened the cardiomyocyte contractile dysfunction.

To show the main cause of cardiac collapse in bupivacaine intoxication, we examined the automaticity, cardiac conduction and contractile response in anesthetized rats. To keep all animals alive until the end of experiment, 9.5 mg/kg of bupivacaine was administered intravenously by slow infusion at a rate of 0.75mg/kg/min, while isoprenalin and adrenalin were given as a bolus. In this model, bupivacaine induced a typical cardiac intoxication. The HR was reduced by more than 30%, while the PR and QRS interval were prolonged by more than 25%. Interestingly, the adrenergic agonist improved the cardiac automaticity but showed no significant effect on cardiac conduction. As shown in Fig 2, adrenalin and isoprenalin significantly increased the HR but showed no effect on the PR and QRS interval.

Bupivacaine-induced cardiac toxicity is not merely a result of blockade of sodium channels, but it also pertains to a direct negative inotropic effect[23]. Bupivacaine-mediated blockade of Nav1.5 channels is characterized by stereo-selectively and a lack of total recovery[24,25]. However, as shown in Fig 1A–1C, the HR, PR interval and QRS interval recovered soon after the termination of the infusion. Consistently, as shown in Fig 5, 101.1 μmol/L bupivacaine totally inhibited the cardiomyocyte contractions. However, washing out the bupivacaine with Tyrode’s solution restored the contractile function. Therefore, we propose that Nav1.5 is not the predominant target of bupivacaine, and some other mechanism(s) must be involved in the cardiac arrest.

The most important finding of our work is that the β-adrenergic activation did not improve, but rather markedly worsened the contractile dysfunction. Both adrenalin and isoprenalin are well known to enhance the LVSP and +dP/dtmax in normal rats. However, as shown in Fig 2A and 2B, adrenalin and isoprenalin mediated a further reduction in the LVSP and +dP/dtmax in our bupivacaine intoxicated rats. Propranolol pretreatment has been reported to reduce the fatal cardiotoxicity due to intravenous bupivacaine in rats, but the underlying mechanism remains unclear[26,27]. Kinney *et al*. proposed that the negative chronotropic effect of propranolol increases tolerance for bupivacaine-induced coronary vasoconstriction, since the reduced compromise of coronary perfusion due to the shortening of diastolic time that is relatively less than that which would occur at a higher heart rate in the absence of propranolol pretreatment[27]. In order to exclude the electrophysiologic and hemodynamic effect of heart disorders, we tested the effect of β-adrenergic activation on bupivacaine-induced contractile dysfunction of freshly isolated single cardiomyocytes. Our data showed that isoprenalin increased the velocity and extent of sarcomere shortening in the presence of bupivacaine lower than 8.9 μmol/L (Fig 4D and 4E), which is consistent with a previous study reporting that adrenalin dysrhythmogenicity was not enhanced by subtoxic bupivacaine[28]. However, in the presence of 20 μmol/L of bupivacaine in this study, isoprenalin induced a further decrease in both velocity and extent of shortening (shown in Fig 4B, 4C, 4E and 4F). As the isoprenalin-induced decrease of cell contraction was inhibited by esmolol, we proposed that isoprenalin would aggravate bupivacaine-mediated cardiac toxicity via the β-adrenergic receptor.

Hiller et al[29]. reported that bupivacaine could accumulate in the myocardium, thereby inducing a reversible mitochondrial swelling, reducing cellular metabolism and consequently causing a negative inotropic effect. In our work, electron microscopy of heart myocardium samples of mice that received bupivacaine showed significant mitochondrial swelling. Isoprenalin significantly increased, while esmolol decreased, the extent of mitochondrial swelling.

It is known that bupivacaine depresses the mitochondrial respiration by inhibiting the activities of respiratory chain complexes I and III, and in turn enhances ROS production[1,30]. In order to show the effect of β-adrenergic activation on bupivacaine-induced depression of the mitochondrial respiration, in this work, ROS production were measured in freshly isolated cardiomyocytes. The cells were electrically stimulated to contract to mimic the situation *in vivo*. Our data showed that bupivacaine increased ROS production in cardiomyocytes. β-adrenergic activation increased the cellular metabolism and therefore mitochondrial oxygen consumption, resulting in a higher rate of mitochondrial ROS production. Consistently, as shown in Fig 9, isoprenalin induced further enhancement in ROS production. Accordingly, we propose that bupivacaine induces cardiac arrest by depressing cardiomyocyte mitochondrial metabolism, and therefore disturbing myofibrillar energy supplementation.

The lack of detailed information on the Na+, K+ and Ca++ currents in bupivacaine-induced cardiotoxicity is the greatest limitation of our work. However, our study convincingly demonstrated that β-adrenergic activation aggravated the bupivacaine-induced cardiac toxicity. The lack of information on ATP production, mitochondrial membrane potential and mitochondrial calcium is another limitation. The effect of β-adrenergic agonist/antagonist on calcium transient in bupivacaine-induced cardiotoxicity will be measured in our future study.

## Conclusion

Our data showed that bupivacaine depressed cardiac automaticity, conductivity and contractility, but the predominant effect was contractile dysfunction which resulted from the disruption of mitochondrial energy metabolism. β-adrenergic activation aggravated the cellular metabolism disorder and therefore contractile dysfunction, this may be the main reason why patients who have undergone bupivacaine-induced cardiac arrest are often resistant to adrenergic therapy.

## Supporting information

S1 Fig Adrenalin and isoprenalin restored bupivacaine reduced HR in SD rats. (TIF)

S2 Fig Dexmedetomidine and esmolol significantly enhanced, whereas isoprenalin significantly reduced the LD50 of bupivacaine in mice. (TIF)

S1 Table Primary data of Figs 1 and 2. Effect of isoprenalin and adrenalin on bupivacain induced cardiac toxicity in vivo.
(XLS)

S2 Table Data of Fig 3. Bupivacaine concentration dependently depressed rats cardiomyocytes sarcomere shortening.
(DOC)

S3 Table Data of Fig 4. Isoprenalin aggravates bupivacaine-induced cardiomyocyte contractile depression.
(DOC)

S4 Table Data of Fig 5. Esmolol inhibits isoprenalin-induced aggravation of myocardial depression.
(DOCX)

S5 Table Data of Fig 6. Washout bupivacaine restore cardiomyocyte contractile capability.
(DOC)

S6 Table Data of Fig 8. Bupivacaine-isoprenalin/esmolol induced mitochondria swelling.
(DOC)

S7 Table Data of Fig 9. Bupivacaine increases ROS production in cardiomyocytes, isoprenalin further increases the production.
(DOCX)

S8 Table Data of LD50 of bupivacaine. (XLS)
