# Supplementary figures and images for: Beta-adrenergic activation induces cardiac collapse by aggravating cardiomyocyte contractile dysfunction in bupivacaine intoxication
Source: PLoS One. 2018 Oct 1;13(10):e0203602. doi: 10.1371/journal.pone.0203602 (PMC6166930; doi:10.1371/journal.pone.0203602)

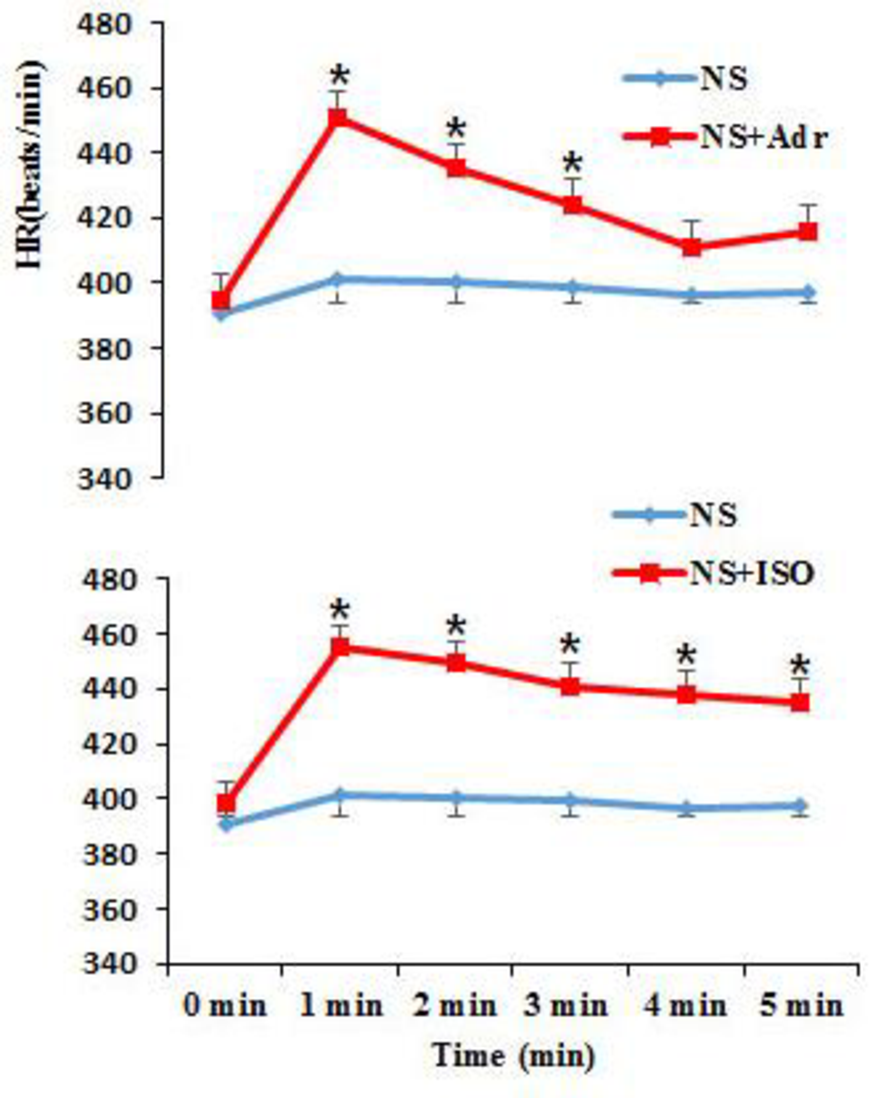

Supplement: S1 Fig — (TIF) [file pone.0203602.s001.tif]
