# Supplementary material for: Beta-adrenergic activation induces cardiac collapse by aggravating cardiomyocyte contractile dysfunction in bupivacaine intoxication
Source: PLoS One. 2018 Oct 1;13(10):e0203602. doi: 10.1371/journal.pone.0203602 (PMC6166930; doi:10.1371/journal.pone.0203602)
Supplement: S4 Table — Esmolol inhibits isoprenalin-induced aggravation of myocardial depression. (DOCX) [file pone.0203602.s006.docx]

**Esmolol inhibits isoprenalin-induced aggravation of myocardial depression**

Method:

Assessment of cell contractility

Shortening of ventricular myocytes was assessed by a video-based edge-detection system (IonOptix, Milton, MA, USA). Briefly, ventricular myocytes were mounted horizontally in an experimental chamber and perfused with normal Tyrode’s solution (1.8 mmol/L CaCl_2_) at 1 ml/min. A pair of electrodes was placed parallel to the cells. Cells were stimulated with 10 volts at a frequency of 0.5 Hz (2-msec duration) using a field stimulator. The real trace of sarcomere shortening, departure velocity of the contraction (dep v), relaxed sarcomere length (basal length, bl) and shortest sarcomere length during the contraction (peak) were recorded. The difference between peak and bl was treated as the sacomere shortening. The percent shortening (bl % peak h) was calculated by using the relaxed sarcomere length as 100%.

The percentage change from base value in the above parameters were tested individually. The test was repeated ten times with different rats, and data are expressed as the mean ± standard deviation (SD). The EC50 of bupivacaine needed to abolish myocyte contraction completely was calculated using Probit analyses. Myocytes were chosen for the study according to the following criteria: (a) rod-shaped appearance with clear striations and no membrane blebs, (b) a negative staircase of twitch performance on stimulation from rest, and (c) absence of spontaneous contractions. Experiments were conducted at room temperature.

Experimental protocol

Freshly isolated cardiomyocytes were perfused with normal Tyrode’s solution until cell contractions stabilized. The perfusate was then changed to Tyrode’s solution containing 13.3 or 20 µmol/L bupivacaine, in the presence of 5.0 nmol/L of isoprenalin, with or without 50.0 nmol/L esmolol to test contractile depression. Thereafter, cells were perfused with normal Tyrode’s solution to wash out the reagents and to ascertain contractile recovery. The contractile parameters were continuously recorded during perfusion

| BP+ISO+E |  |  |  |  |
| --- | --- | --- | --- | --- |
|  |  | bl%peak h(%) | Tp50(%) | dep v(%) |
| 13.3μmol/L | 1 | 117.45 | 126.09 | 105.23 |
|  | 2 | 137.85 | 158.33 | 124.00 |
|  | 3 | 117.93 | 134.78 | 106.99 |
|  | 4 | 107.13 | 128.00 | 121.99 |
|  | 5 | 126.19 | 134.78 | 103.13 |
|  | 6 | 126.42 | 130.43 | 106.48 |
| 20μmol/L | 1 | 105.24 | 155.00 | 96.08 |
|  | 2 | 96.36 | 150.00 | 94.90 |
|  | 3 | 92.15 | 140.00 | 104.09 |
|  | 4 | 99.33 | 150.00 | 101.24 |
|  | 5 | 101.12 | 127.27 | 106.52 |
|  |  |  |  |  |
| BP+ISO |  |  |  |  |
|  |  | bl%peak h(%) | Tp50(%) | dep v(%) |
| 13.3μmol/L | 1 | 88.11 | 169.23 | 107.18 |
|  | 2 | 65.24 | 170.25 | 82.09 |
|  | 3 | 106.11 | 178.57 | 100.41 |
|  | 4 | 92.13 | 161.22 | 68.09 |
|  | 5 | 64.66 | 184.68 | 66.59 |
| 20μmol/L | 1 | 98.63 | 170.00 | 50.80 |
|  | 2 | 58.25 | 183.25 | 85.00 |
|  | 3 | 85.13 | 155.56 | 89.37 |
|  | 4 | 53.84 | 225.00 | 85.45 |
|  | 5 | 68.49 | 185.63 | 55.62 |


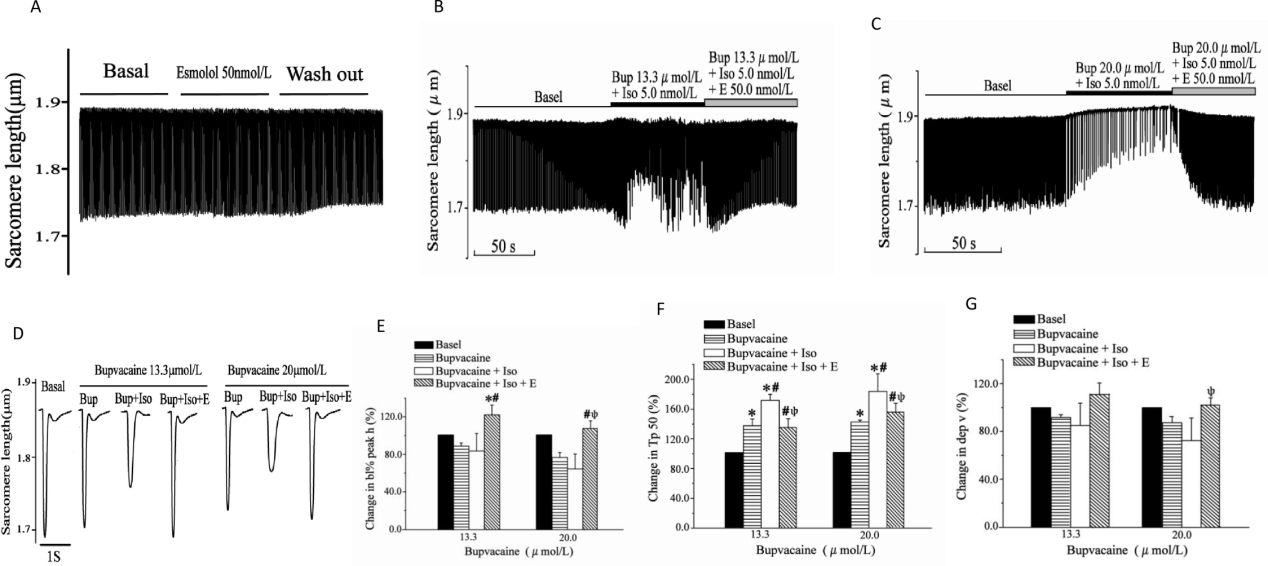


Note:

dep v： Departure velocity of the contraction

Tp50: the time to 50% of the peak speed of myocyte contraction.

bl： Basal length, relaxed sarcomere length

peak：Shortest sarcomere length during the contraction

Sacomere shortening: The difference between peak and bl.

bl % peak h： The percent shortening (bl % peak h) was calculated by using the relaxed sarcomere length as 100%. Or the percentage of the difference between the peak height and baseline values (percentage change from baseline);
