# Supplementary material for: Beta-adrenergic activation induces cardiac collapse by aggravating cardiomyocyte contractile dysfunction in bupivacaine intoxication
Source: PLoS One. 2018 Oct 1;13(10):e0203602. doi: 10.1371/journal.pone.0203602 (PMC6166930; doi:10.1371/journal.pone.0203602)
Supplement: S6 Table — Bupivacaine-isoprenalin/esmolol induced mitochondria swelling. (DOC) [file pone.0203602.s008.doc]

Bupivacaine-isoprenalin/esmolol induced mitochondria swelling

| score | 0 | 1 | 2 | 3 | 4 | 5 |
| --- | --- | --- | --- | --- | --- | --- |
| saline-saline | |  |  |  |  |  |
| 1 | 1 | 18 | 14 | 24 | 12 | 0 |
| 2 | 2 | 18 | 15 | 18 | 6 | 0 |
| 3 | 1 | 15 | 18 | 15 | 6 | 1 |
| 4 | 0 | 13 | 15 | 17 | 12 | 1 |
| 5 | 2 | 16 | 15 | 13 | 4 | 1 |
| 6 | 1 | 17 | 19 | 24 | 11 | 1 |
|  |  |  |  |  |  |  |
| saline-bupivacaine | | |  |  |  |  |
| 1 | 2 | 8 | 13 | 17 | 9 | 1 |
| 2 | 1 | 12 | 16 | 15 | 18 | 1 |
| 3 | 0 | 10 | 10 | 9 | 11 | 3 |
| 4 | 2 | 9 | 14 | 10 | 9 | 3 |
| 5 | 1 | 16 | 13 | 23 | 19 | 1 |
| 6 | 1 | 7 | 14 | 16 | 20 | 2 |
|  |  |  |  |  |  |  |
| Esmolol-bupivacaine | | |  |  |  |  |
| 1 | 1 | 5 | 19 | 20 | 12 | 2 |
| 2 | 0 | 2 | 20 | 9 | 11 | 0 |
| 3 | 0 | 5 | 31 | 12 | 18 | 0 |
| 4 | 0 | 4 | 30 | 16 | 23 | 0 |
| 5 | 0 | 7 | 25 | 21 | 11 | 2 |
| 6 | 0 | 5 | 23 | 22 | 16 | 1 |
|  |  |  |  |  |  |  |
| Isoprenalin-bupivacaine | | |  |  |  |  |
| 1 | 0 | 2 | 24 | 11 | 23 | 7 |
| 2 | 0 | 1 | 6 | 16 | 19 | 2 |
| 3 | 0 | 8 | 30 | 10 | 29 | 0 |
| 4 | 1 | 2 | 5 | 18 | 25 | 11 |
| 5 | 0 | 6 | 6 | 7 | 12 | 8 |
| 6 | 0 | 2 | 6 | 13 | 16 | 6 |

A

B

C

D

Transmission electron microscopy and qualitative morphological scoring

**Drugs and animals**

Male Swiss albino mice (18–24 g) were kept in plastic cages in a room at 25°C under a 12-h light–dark cycle (light on from 7 am to 7 pm). Food and water were available *ad libitum*. Mice were allowed to acclimate to these conditions for ≥3 days after arrival before being used for experiments. All experiments were performed under identical fluorescent lighting between 2 pm and 5 pm.

**Experimental protocol**

Twelve mice were randomly divided into four groups with three mice in each group. Saline, 0.1 mg/kg isoprenalin or 2.0 mg/kg esmolol was injected via the intraperitoneal route at 10 ml/kg body weight. Five minutes after the injection, saline in the control group or 46.7 mg/kg bupivacaine in the other three groups was injected via the intraperitoneal route. The mice were killed by cervical dislocation 3 min after the final injection. The chest was opened, and a tissue block of 1 × 3 mm was taken from the left ventricular wall and fixed with gluteraldehyde at 4°C within 1 min and stored at 4°C.

The tissue was postfixed with 1% osmium tetroxide (pH 7.4) in 0.1 M cacodylate buffer for 1 h at 4°C, and dehydration was performed with ethanol and ethanol-epon. The whole mounts were then epon-embedded overnight at 4°C, and epon-filled flat embedding molds were subsequently polymerized for 48 h at 60°C. Ultrathin sections (60–80 nm) were cut at room temperature with a Leica Ultracut UCT microtome (Leica Microsystems, Wetzlar, Germany) and mounted on nickel grids. Sections were then contrasted with 0.4% lead citrate and 3% uranyl acetate and viewed using a Hitachi H-7500 Transmission Electron Microscope (Hitachi, Tokyo, Japan). All buffers, fixatives and embedding materials for electron microscopy were purchased from the Microscope Center of Hebei Medical University (Shijiazhuang, China).

We evaluated high resolution black and white electron photomicrographs of cardiac mitochondria using the following ultrastructural injury scoring system from 0 to 5, first described by Joshi *et al*.^14^: 0 = normal appearance, 1 = swelling of endoplasmic reticulum, minimal mitochondrial swelling, 2 = mild mitochondrial swelling, 3 = moderate or focal high amplitude swelling, 4 = diffuse high amplitude swelling, disruption of crystal membrane integrity, and 5 = high amplitude swelling with some mitochondrial flocculent densities and/or calcifications. Six photomicrographs of each heart sample depicting between 5 and 10 mitochondria each were evaluated. This scoring was performed by an experimenter blinded to the groups with extensive experience in morphological scoring and electron microscopy.
