# Supplementary material for: Beta-adrenergic activation induces cardiac collapse by aggravating cardiomyocyte contractile dysfunction in bupivacaine intoxication
Source: PLoS One. 2018 Oct 1;13(10):e0203602. doi: 10.1371/journal.pone.0203602 (PMC6166930; doi:10.1371/journal.pone.0203602)
Supplement: S7 Table — Bupivacaine increases ROS production in cardiomyocytes, isoprenalin further increases the production. (DOCX) [file pone.0203602.s009.docx]

Bupivacaine increases ROS production in cardiomyocytes, isoprenalin further increases the production

Methods

The freshly isolated ventricular myocytes from 9 adult SD rat were were plated as triplicates in 24-well plates at a concentration of 5 × 10^5^ cells/well with 500 μl normal Tyrode’s solution (1.8 mmol/L CaCl2) per well, with 8.9 µmol/L or 13.3 µmol/L of bupivacaine, in the presence of 5.0 nmol/L of isoprenalin, with or without 50.0 nmol/L esmolol. A pair of electrodes was placed into the wells. Cells were stimulated with 10 volts at a frequency of 0.5 Hz (2-msec duration) using a field stimulator for 1 min, the intracellular reactive oxygen species levels were evaluated by staining cells with DCFH-DA. The cells were incubated with 10 μM DCFH-DA at 37 °C during the last 20 min treatment. The cells were washed 3 times, collected and resuspended in PBS. Subsequently, the fluorescent signal intensity of DCF was determined by Infinite M1000 PRO premium multimode microplate reader (Tecan Austria GmbH, Grdig, Austria) at an excitation wavelength of 488 nm and at an emission wavelength of 525 nm.

Results:

| **Test 1** |  |  |  |  |
| --- | --- | --- | --- | --- |
| Gain: 85 | well-1 | well-2 | well-3 | mean value |
|  |  |  |  |  |
| DMEM (control) | 3415 | 3751 | 3860 | 3675 |
|  |  |  |  |  |
| 20bupi+iso+esmol | 4957 | 4744 | 4526 | 4742 |
| 20bupi+iso | 4712 | 4829 | 4295 | 4612 |
| 20bupi | 4831 | 4296 | 4467 | 4531 |
|  |  |  |  |  |
|  |  |  |  |  |
| 13.3bupi+iso+esmol | 4022 | 4706 | 4526 | 4418 |
| 13.3bupi+iso | 4647 | 4854 | 4461 | 4654 |
| 13.3bupi | 4253 | 4011 | 4526 | 4263 |
|  |  |  |  |  |
|  | well-1 | well-2 | well-3 | mean |
| 8.9bupi+iso+esmol | 4530 | 4929 | 4371 | 4610 |
| 8.9bupi+iso | 4812 | 5179 | 4292 | 4761 |
| 8.9bupi | 4034 | 3814 | 4380 | 4076 |
|  |  |  |  |  |
|  |  |  |  |  |
|  |  |  |  |  |
| Test 2 |  |  |  |  |
| Gain: 85 |  |  |  |  |
| DMEM (control) | 3273 | 2937 | 2980 | 3063 |
|  |  |  |  |  |
| 20bupi+iso+esmol | 4695 | 4579 | 4736 | 4670 |
| 20bupi+iso | 4619 | 4595 | 4589 | 4601 |
| 20bupi | 3875 | 4093 | 4025 | 3997 |
|  |  |  |  |  |
| 13.3bupi+iso+esmol | 4643 | 4642 | 4956 | 4747 |
| 13.3bupi+iso | 5407 | 5119 | 4574 | 5033 |
| 13.3bupi | 4269 | 4325 | 4279 | 4291 |
|  |  |  |  |  |
| 8.9bupi+iso+esmol | 4718 | 4677 | 5273 | 4889 |
| 8.9bupi+iso | 5282 | 4681 | 5590 | 5184 |
| 8.9bupi | 4287 | 4268 | 4394 | 4316 |
|  |  |  |  |  |
| Test 3 |  |  |  |  |
| Gain: 70 |  |  |  |  |
| DMEM (control) | 3815 | 4103 | 3827 | 3915 |
|  |  |  |  |  |
| 20bupi+iso+esmol | 5118 | 6510 | 5817 | 5815 |
| 20bupi+iso | 4684 | 5003 | 5697 | 5128 |
| 20bupi | 4097 | 4304 | 5279 | 4560 |
|  |  |  |  |  |
|  |  |  |  |  |
| 13.3bupi+iso+esmol | 6248 | 5890 | 6420 | 6186 |
| 13.3bupi+iso | 4743 | 5949 | 5717 | 5469 |
| 13.3bupi | 4272 | 4348 | 5258 | 4626 |
|  |  |  |  |  |
| 8.9bupi+iso+esmol | 6757 | 5982 | 5474 | 6071 |
| 8.9bupi+iso | 5702 | 6432 | 6743 | 6292 |
| 8.9bupi | 5614 | 4907 | 5019 | 5180 |
|  |  |  |  |  |
|  |  |  |  |  |
| Test 4 |  |  |  |  |
| Gain: 90 |  |  |  |  |
| DMEM (control) | 4205 | 3790 | 4133 | 4042 |
|  |  |  |  |  |
| 20bupi+iso+esmol | 4675 | 4685 | 4784 | 4714 |
| 20bupi+iso | 4680 | 3867 | 4654 | 4400 |
| 20bupi | 4868 | 4100 | 4569 | 4512 |
|  |  |  |  |  |
|  |  |  |  |  |
| 13.3bupi+iso+esmol | 4748 | 3730 | 4421 | 4299 |
| 13.3bupi+iso | 4849 | 4709 | 5230 | 4929 |
| 13.3bupi | 4683 | 4385 | 4132 | 4400 |
|  |  |  |  |  |
| 8.9bupi+iso+esmol | 4958 | 5082 | 4644 | 4894 |
| 8.9bupi+iso | 5819 | 5565 | 4910 | 5431 |
| 8.9bupi | 4630 | 4637 | 4943 | 4736 |
|  |  |  |  |  |
|  |  |  |  |  |
| Test 5 |  |  |  |  |
| Gain: 90 |  |  |  |  |
| DMEM (control) | 3924 | 4317 | 4180 | 4140 |
|  |  |  |  |  |
| 20bupi+iso+esmol | 4833 | 4607 | 4689 | 4709 |
| 20bupi+iso | 4603 | 4759 | 4814 | 4725 |
| 20bupi | 4406 | 4756 | 8206 | 5789 |
|  |  |  |  |  |
| 13.3bupi+iso+esmol | 4921 | 4870 | 4760 | 4850 |
| 13.3bupi+iso | 5714 | 5610 | 4850 | 5391 |
| 13.3bupi | 4925 | 4534 | 4597 | 4685 |
|  |  |  |  |  |
| 8.9bupi+iso+esmol | 5815 | 5336 | 5082 | 5411 |
| 8.9bupi+iso | 5368 | 5648 | 4941 | 5319 |
| 8.9bupi | 4861 | 4871 | 4951 | 4894 |
|  |  |  |  |  |
| Test 6 |  |  |  |  |
|  |  |  |  |  |
| Gain: 80 |  |  |  |  |
| DMEM (control) | 5246 | 5057 | 5198 | 5167 |
|  |  |  |  |  |
| 20bupi+iso+esmol | 5260 | 5663 | 5582 | 5501 |
| 20bupi+iso | 5050 | 4855 | 5434 | 5113 |
| 20bupi | 5831 | 5180 | 5369 | 5460 |
|  |  |  |  |  |
| 13.3bupi+iso+esmol | 5694 | 5864 | 6317 | 5958 |
| 13.3bupi+iso | 6321 | 6790 | 5871 | 6327 |
| 13.3bupi | 5344 | 5439 | 5885 | 5556 |
|  |  |  |  |  |
| 8.9bupi+iso+esmol | 6513 | 5795 | 6262 | 6190 |
| 8.9bupi+iso | 5586 | 5755 | 5840 | 5727 |
| 8.9bupi | 5529 | 5529 | 5498 | 5518 |
|  |  |  |  |  |
| Test 7 |  |  |  |  |
| Gain: 85 |  |  |  |  |
| DMEM (control) | 4019 | 4056.8 | 4102 | 4059 |
|  |  |  |  |  |
| 20bupi+iso+esmol | 5363 | 5153 | 5217 | 5244 |
| 20bupi+iso | 4947 | 5134 | 4860 | 4980 |
| 20bupi | 4735 | 4938 | 4903 | 4858 |
|  |  |  |  |  |
| 13.3bupi+iso+esmol | 5450 | 5366 | 5447 | 5421 |
| 13.3bupi+iso | 5789 | 5953 | 6138 | 5960 |
| 13.3bupi | 5235 | 5273 | 5344 | 5284 |
|  |  |  |  |  |
| 8.9bupi+iso+esmol | 5902 | 5805 | 5614 | 5773 |
| 8.9bupi+iso | 5910 | 6151 | 6213 | 6091 |
| 8.9bupi | 5588 | 5360 | 5499 | 5482 |
|  |  |  |  |  |
| Test 8 |  |  |  |  |
| Gain: 95 |  |  |  |  |
|  |  |  |  |  |
| DMEM (control) | 3518 | 3610 | 3360 | 3496 |
|  |  |  |  |  |
| 20bupi+iso+esmol | 3980 | 4210 | 4092 | 4094 |
| 20bupi+iso | 3964 | 4353 | 4445 | 4254 |
| 20bupi | 3862 | 4199 | 3878 | 3979 |
|  |  |  |  |  |
| 13.3bupi+iso+esmol | 4608 | 4251 | 4736 | 4531 |
| 13.3bupi+iso | 5057 | 4614 | 4968 | 4879 |
| 13.3bupi | 4008 | 4509 | 3795 | 4104 |
|  |  |  |  |  |
| 8.9bupi+iso+esmol | 4243 | 4430 | 4144 | 4272 |
| 8.9bupi+iso | 4613 | 4790 | 4586 | 4663 |
| 8.9bupi | 3908 | 3832 | 4014 | 3918 |
|  |  |  |  |  |
| Test 9 |  |  |  |  |
| Gain: 90 |  |  |  |  |
|  |  |  |  |  |
| DMEM (control) | 3080 | 3237 | 2617 | 2978 |
|  |  |  |  |  |
| 20bupi+iso+esmol | 3503 | 3706 | 3448 | 3552 |
| 20bupi+iso | 4230 | 5230 | 4196 | 4552 |
| 20bupi | 3108 | 3216 | 3382 | 3235 |
|  |  |  |  |  |
| 13.3bupi+iso+esmol | 3368 | 3901 | 3350 | 3539 |
| 13.3bupi+iso | 3995 | 3743 | 3905 | 3881 |
| 13.3bupi | 3345 | 3065 | 3324 | 3244 |
|  |  |  |  |  |
| 8.9bupi+iso+esmol | 3665 | 4012 | 3973 | 3883 |
| 8.9bupi+iso | 4077 | 4435 | 4205 | 4239 |
| 8.9bupi | 3568 | 3248 | 3478 | 3431 |

Note：

8.9bupi+iso+esmol: 8.9 µmol/L bupivacaine +5.0 nmol/L isoprenalin+50.0 nmol/L Esmolol

8.9bupi+iso: 8.9 µmol/L bupivacaine+50.0 nmol/L isoprenalin

8.9bupi: 8.9 µmol/L bupivacaine

13.3bupi+iso+esmol: 13.3 µmol/L bupivacaine+5.0 nmol/L isoprenalin+50.0 nmol/L Esmolol

13.3bupi+iso: 13.3 µmol/L bupivacaine+5.0 nmol/L isoprenalin

13.3bupi: 13.3 µmol/L bupivacaine

20bupi+iso+esmol: 20 µmol/L bupivacaine+5.0 nmol/L isoprenalin+50.0 nmol/L Esmolol

20bupi+iso: 20µmol/L bupivacaine+5.0 nmol/L isoprenalin

20bupi: 20 µmol/L bupivacaine
